# Strengthening clinical cancer research in the United Kingdom

**DOI:** 10.1038/bjc.2011.69

**Published:** 2011-03-01

**Authors:** M Stead, D Cameron, N Lester, M Parmar, R Haward, R Kaplan, T Maughan, R Wilson, H Campbell, R Hamilton, D Stewart, L O'Toole, D Kerr, V Potts, R Moser, J Darbyshire, P Selby

**Affiliations:** 1 National Institute for Health Research Clinical Research Network Coordinating Centre, Leeds, UK; 2 National Cancer Research Network Coordinating Centre, Leeds, UK; 3 Department of Oncology, University of Edinburgh, Edinburgh, UK; 4 National Cancer Research Network Coordinating Centre, London, UK; 5 Medical Research Council Clinical Trials Unit, London, UK; 6 School of Medicine, Cardiff University, Cardiff, UK; 7 Wales Cancer Trials Network Coordinating Centre, Cardiff, UK; 8 N. Ireland Cancer Clinical Trials Unit, Belfast, Northern Ireland; 9 Department of Health, England, UK; 10 National Cancer Research Institute, London, UK; 11 Arthritis Research UK, London, UK; 12 Department of Clinical Pharmacology, University of Oxford, Oxford, UK; 13 National Translational Cancer Research Network, Oxford, UK

**Keywords:** clinical trials, patient recruitment

## Abstract

**Background:**

In 1999, 270 000 cases of cancer were registered in the United Kingdom, placing a large burden on the NHS. Cancer outcome data in 1999 suggested that UK survival rates were poorer than most other European countries. In the same year, a Department of Health review noted that clinical trials accrual was poor (<3.5% of incident cases) and hypothesised that increasing research activity might improve outcomes and reduce the variability of outcomes across England. Thus, the National Cancer Research Network (NCRN) was established to increase participation in cancer clinical research.

**Methods:**

The NCRN was established in 2001 to provide a robust infrastructure for cancer clinical research and improvements in patient care. Remit of NCRN is to coordinate, support and deliver cancer clinical research through the provision of research support staff across England. The NCRN works closely with similar networks in Scotland, Wales and the Northern Ireland. A key aim of NCRN is to improve the speed of research and this was also assessed by comparing the speed of study delivery of a subset of cancer studies opening before and after NCRN was established.

**Results:**

Patient recruitment increased through NCRN, with almost 32 000 (12% of annual incident cases) cancer patients being recruited each year. Study delivery has improved, with more studies meeting the recruitment target – 74% compared with 39% before NCRN was established.

**Conclusion:**

The coordinated approach to cancer clinical research has demonstrated increased accrual, wide participation and successful trial delivery, which should lead to improved outcomes and care.

In 1999, 270 000 new cases of cancer were registered in the United Kingdom, placing a large burden on the NHS. Data published in 1999 (Berrino *et al*, 1999) suggested that survival rates were poorer in the United Kingdom than in most other European countries. In the same year, a review by the Department of Health, England (Cancer Working Group, 1999) noted that accrual into clinical trials was poor (<3.5% of incident cases) and hypothesised that increasing research activity might improve outcomes and reduce the variability of outcomes across the country. These findings were instrumental in the establishment of the National Cancer Research Network (NCRN).

It is accepted that prospective randomised controlled trials (RCTs), supported by other well-designed clinical studies, provide the evidence base for the provision of excellent healthcare and the basis for future developments. The benefits for patients from participating in clinical research are therefore based substantially on the provision of an excellent evidence base for high-quality healthcare. However, there has been considerable discussion about whether there are benefits from clinical research, which arise other than from the implementation of the results of high quality trials and studies. This topic has been reviewed (Braunholtz *et al*, 2001; Peppercorn *et al*, 2004; Vist *et al*, 2008). There is little convincing evidence that an individual patient when treated in the context of a clinical trial within a particular healthcare institution or healthcare system will have better health outcomes than a similar patient receiving the same treatment as standard care in the same system. However, there is stronger support in the literature for the hypothesis that healthcare systems which are research active rather than those which are not have better health outcomes perhaps through the introduction of state-of-the-art care approaches. This latter question has been addressed in relatively few studies (Karjalainen and Palva, 1989; du Bois *et al*, 2005; Majundar *et al*, 2008), all of which provide supportive evidence.

Here, we provide an overview of the impact of the NCRN and its UK partner Research Networks since 2001, including a comparison of study delivery before and after NCRN was established.

## Materials and methods

### Establishment of NCRN and its Partner Research Networks

NCRN was established in 2001 by the Department of Health, England and managed by a consortium of the University of Leeds and the Medical Research Council Clinical Trials Unit. It is now part of the National Institute for Health Research (NIHR).

The aims of NCRN are to:

- Improve patient care by speeding up access to the best treatment and care for people in all parts of the country.
- Improve the coordination of research by providing an effective and efficient mechanism for conducting research in areas that have been agreed as priorities.
- Improve the speed of research by increasing the numbers of patients in research and the rate at which they are recruited.
- Maintain and enhance research quality by providing the processes necessary to develop high-quality research protocols and the infrastructure necessary to carry them out.
- Widen participation in the research by increasing the number of NHS organisations, healthcare professionals and patients participating in research studies.

The initial target was to double the number of adult cancer patients who entered into clinical research studies in 3 years to 15 000 (7.5%) patients.

The NCRN in England works closely with other cancer research networks across the United Kingdom, each of which is funded by its respective health department, and with differing approaches to the portfolio of trials supported. The Wales Cancer Trials Network, comprising three Research Networks, was established in 1998. In 2002, the Scottish Cancer Research Network involving three Research Networks was established. The Northern Ireland Cancer Trials Network was established in 2007.

Patients and the public have been actively involved to ensure that the voice of people affected by cancer helped form, inform and reform the cancer research.

As part of NCRN, 34 Research Networks were established across England, each with a Clinical Lead and Network Manager, plus £200 000 per one-million catchment population. Routine care of cancer patients was already organised into Cancer Services Networks that reflected clinical care pathways, and each Research Network mapped onto these Networks. The organisation of the Research Networks varies depending on local factors, including leadership, size, geography, demography and service organisation, and existing research infrastructure including the presence or absence of medical schools and teaching hospitals. Wherever possible, NCRN staff are fully integrated with existing research staff and with clinical staff in their local Cancer Services Network.

The NCRN has developed into a comprehensive, nationwide, inclusive organisation, integral to Cancer Services and forming links with every hospital in the country (from large university teaching hospitals to small district general hospitals), with growing links to primary care, and with all disciplines involved in the research process.

The majority of Network spend is on staffing. There are now over 750 Network-funded research support staff (528 whole-time equivalent staff), half of which are Research Nurses.

NCRN has also developed close working relationships with all organisations involved in the funding, sponsorship and management of cancer research – charities, Clinical Trial Units, patient/public groups and industry.

### Comparison of recruitment into studies that closed before and after NCRN was established

Two groups of non-commercial multicentre RCTs were identified to compare trial delivery before and after NCRN was established. The United Kingdom Coordinating Committee on Cancer Research Trial Register, a comprehensive database of all cancer RCTs, was searched to identify RCTs that closed before NCRN was established. The search identified 37 RCTs, of which nine recruited most patients from non-UK sites and were excluded. The pre-NCRN group included 28 RCTs that closed between 1996 and 2000.

Studies for the ‘post-NCRN’ period included studies in the NCRN Portfolio Database that closed to recruitment in 2005, 2006 or 2007 and had been funded by Cancer Research UK, the Medical Research Council or Department of Health. This identified 40 studies, of which nine recruited most patients from non-UK sites and were excluded. The post-NCRN group included 31 RCTs.

Factors analysed included whether or not studies met their planned recruitment target, and whether or not the speed of trial recruitment was greater after NCRN.

## Results

Data are presented on the national portfolio of cancer research studies and accrual for the period 1 April 2001 to 31 March 2008, and on the impact of the NCRN on study delivery.

### Portfolio development

The NCRN portfolio was established to maximise the impact of the NCRN in improving patient care by ensuring that NCRN infrastructure was used to support high-quality research relevant to the needs of the NHS. It incorporates all larger Phase II and Phase III trials, together with other well-designed studies, that were funded by the NIHR and its partners, plus a number of other studies (e.g., international studies). In 2005, the NCRN's remit was extended to include industry-funded studies.

To ensure the development of nationwide portfolios, several UK-wide Clinical Studies’ Groups were established, in some cases building on existing structures. Each Group has multidisciplinary scientific members, patient/public representatives and funding body representatives. The Groups have a central role in the development and oversight of a balanced national portfolio and are the preferred, and in practice now often the primary, route for study development. The Clinical Studies’ Groups focus either on specific cancer types or on generic groups, for example, primary care. All proposed studies emerging from these groups are still required to secure their research funding via the usual competitive routes before they can open within the NCRN portfolio.

The number of studies has more than doubled since NCRN was established, with a steady growth in both randomised and non-randomised studies. Table 1 shows the number of studies open to recruitment or in set-up in each financial year.

### Accrual

#### Overall accrual

**Figure 1:**
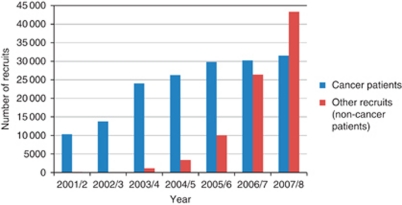
Annual UK accrual to NCRN portfolio studies.

The initial target of doubling the number of adult cancer patients entered into clinical research studies in England within 3 years was met ahead of schedule. Figure 1 shows the total annual NCRN UK accrual. Collection of accrual data for other recruits (i.e., not cancer patients) into screening and prevention studies was not done routinely until 2004/5.

Accrual of cancer patients increased steadily in the first 5 years, with increased recruitment to both randomised and non-randomised studies. Although there has been a slowing in the rate of increase in recruitment since 2005/6 in terms of cancer patients, significant NCRN resources are now being used in screening and prevention studies with over 43 000 other individuals being recruited to these studies in 2007/8. ^*^Web link to detailed data in Appendix Table A1.

**Figure 2:**
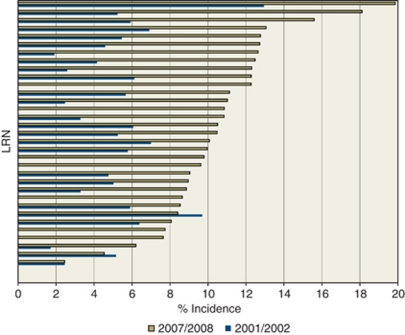
Accrual as a percentage of annual cancer incidence in each Local Research Network (LRN) in England for 2001/2 and 2007/8.

Recruitment by Local Research Network varied considerably. Figure 2 presents the percentage accrual incidence for each of the 34 Local Research Networks in England for 2001/2 and 2007/8. We explored whether the type of Network affected the level of activity by clustering Networks according to the presence of major teaching or research centres, the resources available outside NCRN funding, the size of the Network, the number and type of NHS Trusts in the Network and the presence of a wider research infrastructure, but found no evidence of different impacts.

In 2001/2, only two Local Research Networks recruited the NCRN target of 7.5% of incident patients, and only one Network recruited more than 10% of patients. By 2007/8, almost every Network had reached the target of 7.5%, with over half of the Networks (18/32) entering more than 10% of patients into high-quality cancer research studies.

Patient accrual increased for nearly all cancer types, including common and less common cancers. In 2001/2, only six patients with head and neck cancer were recruited into high-quality national studies, compared with 163 in 2007/8. Recruitment in breast cancer increased from 4011 patients in 2001/2 to 11 401 patients in 2007/8.

More clinical sites were involved in cancer research through NCRN, demonstrating increased access to studies for patients. Across the United Kingdom, 66 Trusts have radiotherapy facilities and these centres generally provide expertise in the management of all cancers within their immediate geographical locality and of less common cancers referred from other hospitals. There was an overall increase in recruitment from all centres; recruitment from centres with radiotherapy facilities doubled and recruitment from centres without radiotherapy facilities almost quadrupled. The centres without radiotherapy facilities, generally district hospitals, are now collectively contributing almost half of the patients entered into all NCRN studies, demonstrating wider participation in research studies as a result of the NCRN support.

### Recruitment into studies that closed before and after NCRN was established

#### Proportion that achieved the recruitment target

**Figure 3:**
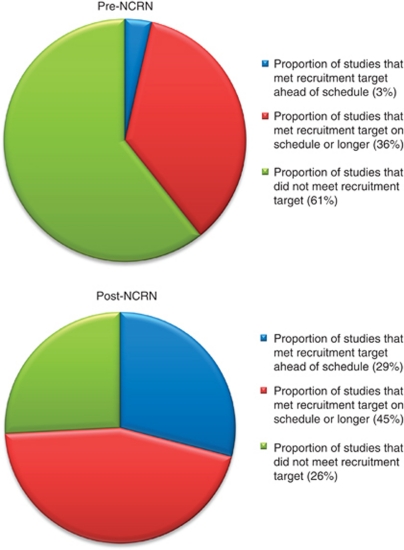
Proportion of studies meeting the study's recruitment target.

Figure 3 shows the proportion of studies that met the recruitment target (irrespective of time frames) for the subsets of studies that closed before and after NCRN was established. In all, 17 of 28 studies (61%) closing before NCRN was established did not meet their recruitment target. Of these 17 studies, 7 recruited >90%, 4 recruited 50–80% and 6 recruited <20% of the planned target. In comparison, only 8 of 31 studies (26%) closing after the establishment of NCRN did not meet their planned recruitment target. Of these eight studies, one recruited >90%, one recruited 50–80%, four recruited 20–49% and two recruited <20% of the planned target. After NCRN was established, 29% of studies met the recruitment target ahead of schedule, compared with only 3% of studies that closed before NCRN was established.

#### Speed of recruitment.

**Figure 4:**
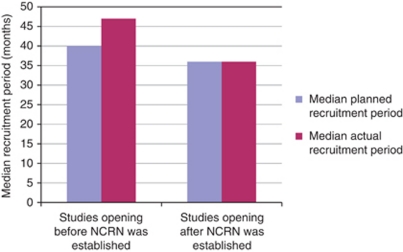
Comparison of median planned and actual recruitment periods for studies opening to recruitment before and after NCRN was established.

Figure 4 shows that the median actual recruitment period for studies that closed before NCRN was established was longer than the planned period, by an average of 7 months, whereas the median actual recruitment for studies in the post-NCRN period was the same as the planned recruitment period.

#### Comparison of sample sizes and actual number of patients recruited.

**Figure 5:**
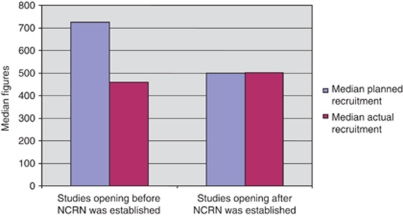
Comparison of median planned and actual recruitment figures for studies opening to recruitment before and after NCRN was established.

Figure 5 shows the median planned recruitment figures compared with the median actual recruitment figures for studies closing before NCRN was established and for studies closing after NCRN was established.

Figure 5 shows that, on average, the number of patients recruited to studies in the pre-NCRN period was significantly less than the planned sample size, but after NCRN was established, target sample sizes were usually met.

The data in Figures 3, 4, 5 show a general trend towards improvement in terms of delivery of cancer research studies.

## Discussion

The coordinated and managed approach to clinical research described here has demonstrated increased accrual and faster trial delivery, which should improve outcomes and care. The United Kingdom accrual of cancer patients to clinical studies has increased since the establishment of NCRN, with almost 32 000 (12% of annual incident cases) cancer patients recruited in 2007/8 compared with 27 012 patients entering into similar National Cancer Institute US studies in October 2006–September 2007 and with 5473 patients entering into European Organisation for Research and Treatment of Cancer studies in 2007. Having a clear national target provided a focus for the delivery of studies in a short period of time.

Integration of the Research Networks with the Cancer Service Networks from NCRN's initiation provided a structure for collaborative working and placed clinical research on the agenda of service provision. The provision of over 700 dedicated and funded personnel focussing on the support and delivery of cancer clinical research has also contributed to the success of the NCRN. National coordination of the research support staff streamlined and standardised human resources systems such as job descriptions and provided consistent high-quality training and development.

The portfolio has grown in nearly all cancers, with increases in both randomised and non-randomised studies. Although the portfolio now includes more screening and prevention studies and more genetic epidemiology studies, the majority of studies remain interventional drug trials, and ongoing work is needed to encourage the development of other types of interventional study, such as new medical devices, surgical and radiotherapeutic techniques. The success of the NCRN approach to support clinical cancer research suggests that this coordinated and resourced enhancement in recruitment could be harnessed to preferentially support portfolio development and recruitment into non-drug trials and rare cancer sites, although not withdrawing support from important studies in the more common cancers. Future developments of the NCRN are to move towards a more balanced portfolio in terms of common and rare cancers, drug and non-drug trials, and academic or commercially sponsored studies.

Increases in accrual across the whole of the United Kingdom demonstrate a broadening of access to research studies, with patients more often being able to enter studies at their local hospital, whether or not it is a teaching hospital, facilitating recruitment across the whole of the country and thus reducing geographical inequalities in access to clinical research.

In 2001/2, only two Local Research Networks recruited more than 7.5% of patients. By 2007/8, almost every Network had reached the target of 7.5% of patients, with over half of the Networks recruiting more than 10% of patients. We explored whether the type of Network affected the level of activity, but found no consistent pattern; this target was reached ahead of schedule and indeed exceeded, demonstrating that a coordinated and targeted effort can result in increased recruitment, geographically broader trial participation and timely delivery of research studies, while maintaining high standards of research quality. Cancer patient outcomes improved steadily during the implementation of the NCRN (Rachet *et al*, 2009), but it is too early to determine how much NCRN contributed to this. The anticipated contribution will depend on the implementation of positive trial results and probably on increased participation (du Bois *et al*, 2005; Majundar *et al*, 2008) and the number of hospitals participating in cancer clinical research.

We believe that the use of this simple target, combined with the commitment, drive and motivation of the Clinical Lead, the Research Network Manager and the clinicians, and research support staff within the Network, encouraged broad support from the local NHS leading to increases in the levels of research activity.

Although recruitment increased steadily in the first 5 years of NCRN, the rate of growth has slowed in the past 2 years. This is likely to be due to a number of factors, including networks reaching saturation point with their current resources. Increasing numbers of patients are on follow-up (estimated to be 84 000 patients in 2006/7), placing an increased burden on the networks. In addition, the EU Directive for Clinical Trials was translated into UK Law in 2004 and resulted in trials of medicinal products taking longer to open as a result of increased regulatory approvals and documentation and uncertainty about different elements of its implementation (Hearn and Sullivan, 2007).

As well as increasing the levels of activity and broadening study participation, study delivery has improved. More studies open to recruitment during NCRN met the recruitment target – 74% compared with 39% for studies closing before NCRN was established. This is also higher than that noted in a review of trials that had started in or after 1994 and were due to end before 2003, where only 31% of the 114 RCTs funded by the Medical Research Council or Health Technology Assessment Programmes in a range of diseases recruited successfully (Campbell *et al*, 2007). After the establishment of NCRN, recruitment to time and target was achieved more often. These data suggest a trend towards improvement in terms of delivery of cancer research studies, although there is still scope for improvement in individual studies.

The success of NCRN has been a major driver in the establishment of the NIHR Clinical Research Network, which aims to support all areas of clinical research, including industry-funded commercial trials, and to facilitate the conduct of trials and other well-designed studies across the United Kingdom. The Department of Health is investing in improving the research management and governance procedures to speed up the approval of studies.

## Figures and Tables

**Table 1:** Numbers of UK cancer studies open to recruitment or in set-up in each financial year

	**2001/02**	**2002/03**	**2003/04**	**2004/05**	**2005/06**	**2006/07**	**2007/08**
Total number of studies	182	240	304	337	368	414	451
Number of randomised studies	124	152	184	197	217	252	271
Number of non-randomised studies	58	88	120	140	151	162	180

**Table A1:** Detailed annual accrual of cancer patients into NCRN portfolio studies

	**All**	**2001/02**	**2002/03**	**2003/04**	**2004/05**	**2005/06**	**2006/07**	**2007/08**
	**Cancer**	**Total**	**Total**	**Total**	**Total**	**Total**	**Total**	**Total**
	Randomised	8141	10 419	12 392	13 166	10 801	9728	11 079
	Non-randomised	1943	2722	9470	10 587	15 183	16 787	16 357
England total	10 084		13 141	21 862	23 753	25 984	26 515	27 436
								
	Randomised			43	172	83	69	95
	Non-randomised			19	119	169	166	113
Northern Ireland total				62	291	252	235	208
								
	Randomised	134	279	901	1151	1136	1241	1111
	Non-randomised	0	19	331	256	796	600	676
Scotland total		134	298	1232	1407	1932	1841	1787
								
	Randomised	118	243	594	586	602	592	808
	Non-randomised	20	97	277	226	1017	1095	1525
Wales total		138	340	871	812	1619	1687	2333
								
NCRN total	Randomised	8161	10 941	13 930	15 075	12 622	11 630	13 093
	Non-randomised	2195	2838	10 097	11 188	17 165	18 648	18 671
NCRN total		10 356	13 779	24 027	26 263	29 787	30 278	31 764

Abbreviation: NCRN=National Cancer Research Network.

## APPENDIX

Table A1
